# A Lightweight and High Yield Complementary Metal-Oxide Semiconductor True Random Number Generator with Lightweight Photon Post-Processing

**DOI:** 10.3390/s24237502

**Published:** 2024-11-25

**Authors:** Chi Trung Ngo, Hyun Woo Ko, Ji Woo Choi, Jae-Won Nam, Jong-Phil Hong

**Affiliations:** 1 School of Electrical Engineering, Chungbuk National University, Cheongju 28644, Republic of Korea; trung@cbnu.ac.kr (C.T.N.); khw@cbnu.ac.kr (H.W.K.); sb7429@cbnu.ac.kr (J.W.C.); 2 Department of Electronic Engineering, Seoul National University of Science and Technology, Seoul 01811, Republic of Korea; jaewon.nam@seoultech.ac.kr

**Keywords:** lightweight cryptography, true random number generator, photon, IoT

## Abstract

This paper introduces a novel TRNG architecture that employs a wave converter to generate random outputs from the jitter noise in a customized ring oscillator (RO). Using a current-starved inverter, the proposed RO offers the option of operating three different oscillation frequencies from a single oscillator. To assess its performance, the core TRNG proposed in this work was designed with multiple samples, employing various transistor sizes for 28 nm CMOS processes. The measurements show that only a small number of measured TRNG samples passed the randomness NIST SP 800-22 tests, which is a common problem, not only with the proposed TRNG but also with other TRNG structures. To solve this issue, a lightweight post-processing algorithm using the Photon hash function was newly applied to the proposed TRNGs topology. The lightweight Photon hash function-based post-processing was implemented with the proposed TRNG topology in a 28 nm CMOS process. The design occupies 16,498 µm^2^, with a throughput of 0.0142 Mbps and power consumption of 31.12 mW. Measurements showed significant improvement, with a 50% increase in chips passing the NIST SP 800-22 tests. Compared with the conventional DRBG post-processing method, the proposed lightweight Photon post-processing reduces area occupation by five times and power consumption by 65%.

## 1. Introduction

In internet of thing (IoT) networks, IoT devices collect important data and transmit them through a wireless communication network. As data transmission through wireless communication is an insecure channel, important data from IoT devices must be encrypted and transmitted using a cryptographic algorithm to prevent hacking [1,2]. In the realm of cryptography, random number generators (RNGs) play a crucial role in generating encryption keys and initialization vectors for authentication, integrity, confidentiality, and digital signature [3].

True random number generators (TRNGs) generate random numbers from a source of entropy or unpredictability in the physical resources that are not generated by algorithms or mathematical formulas. TRNGs based on electronic systems using the CMOS process are suitable for IoT devices due to their low cost, low power consumption, and high integration density. Several TRNG structures based on the CMOS process have been reported, and the most widely used methods include using noise source amplification [4], metastable behavior [5], and jitter from oscillators [6,7,8]. The noise source amplification method leverages the inherent randomness of electronic noise, specifically thermal noise, generated by components like resistors or transistors in CMOS circuits. This technique involves extracting and amplifying this noise to create a truly random binary output. The initial step is to capture the noise, often through amplification with operational amplifiers or dedicated CMOS amplifiers, since the noise source itself tends to be weak. Subsequently, the amplified signal is digitized using analog-to-digital converters, and optional post-processing methods may be employed to enhance the quality and uniformity of the random numbers. Therefore, the noise source amplification method consumes more power and requires complex circuitry compared to other TRNG methods. The metastability method creates the random number by using a metastable state that is a highly unpredictable state in digital flip-flop or latch [5,9]. However, this method necessitates extensive calibration and post-processing to mitigate bias introduced by transistor mismatch. This requirement increases area occupation of the system. Conventional jitter-based TRNGs are a type of hardware random number generator that exploits the inherent variability in signal timing or clock oscillations to generate truly random numbers. Jitter refers to small, unpredictable variations in the timing of digital signals, which are used to extract random bits. However, this approach typically results in relatively low entropy and poor randomness due to limited variations in the oscillation frequencies of the ROs. Recently, several methods have been published to improve randomness, including edge-chasing-based TRNGs [10] and self-timed, ring-based TRNGs [11]. However, compared to conventional ring oscillator (RO) structures, the edge-chasing-based TRNG described in [10] necessitates costly calibration in terms of area occupation to solve digital layout mismatch and process variation. The self-timed ring structure demands multiple states to achieve an acceptable level of randomness. Specifically, as noted in [11], 45 states are necessary to attain state-of-the-art performance, which increases the area occupation.

NIST SP 800-22 [12] and DIEHARD [13] provide a suite of statistical tests to evaluate the quality of TRNGs. These tests check for various statistical properties of the generated sequence, such as uniformity, independence, and randomness, rather than their entropy. The sophisticated standard statistical tests are complex, expensive, slow, and rely on extensive datasets. When evaluating the performance of TRNGs, it is crucial to apply a large number of TRNG samples to ensure the reliability of the measurements. Due to the environmental variations in the entropy source of TRNG chips [14], it is difficult to guarantee a large number of chips passing high-end standard statistical tests, causing yield degradation problems and making commercialization difficult. To address this issue, a lightweight conditioner (post-processing algorithm) is required to enhance the statistical quality of the output [12]. This conditioner must be compact enough to meet the constraints of resource-limited devices, thereby improving the design’s commercial viability. Furthermore, the quality of the TRNG architecture must be validated through testing with a large number of samples to ensure the performance and robustness.

This work proposes a novel CMOS-based RO-TRNG with a wave converter and a lightweight post-processing method that significantly improves the yield problem to satisfy NIST SP 800-22. Additionally, the proposed RO-TRNG can operate three different oscillation frequencies in a single core oscillator by controlling the current-starved switching transistors. Section 2 introduces the topology and operating principle of the proposed RO-based TRNG with a wave converter. Comprehensive details on the post-processing algorithms candidates and the selection of the proposed lightweight Photon-based methods are provided in Section 3. Section 4 presents the implemented architecture of the overall proposed TRNG architecture. The measurement results of the implemented TRNG are discussed in Section 5, and finally, the paper concludes in Section 6.

## 2. Proposed CMOS RO-TRNG with Wave Converter

**Figure 1:**
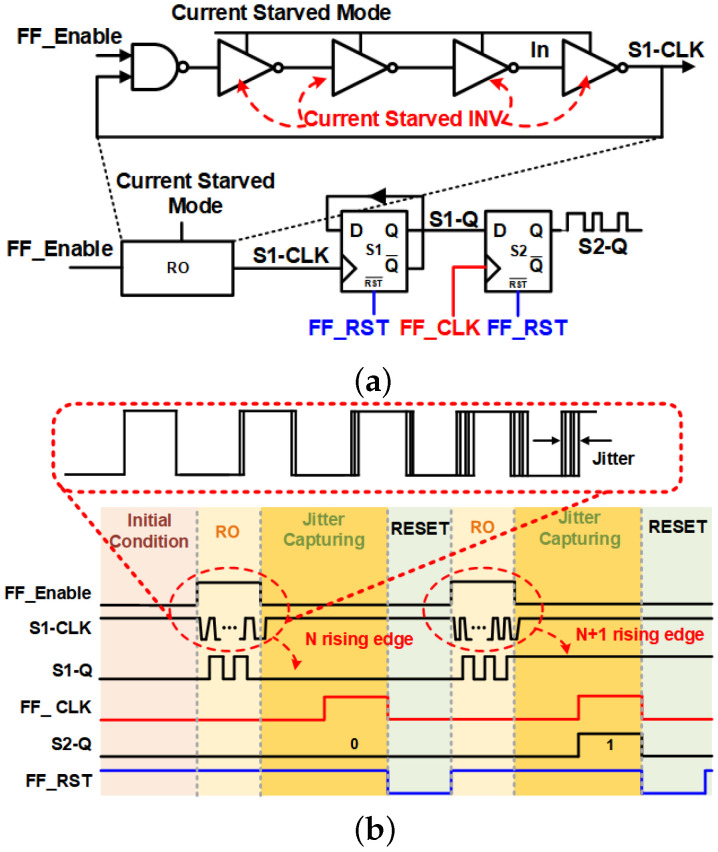
(**a**) Schematic of the wave converter RO TRNG and (**b**) its timing diagram.

The proposed TRNG utilizes the jitter from a single RO by employing a wave converter. As illustrated in Figure 1a, the proposed TRNG adopts only one RO as a source of randomness and two DFFs as the wave converter. The selection of the RO structure for the TRNG design was motivated by its inherent simplicity and portability. Conventional RO-based TRNG designs often encounter challenges related to low entropy and poor randomness because of the limited variations in the oscillation frequencies of a single RO. Attempts to enhance TRNG quality by incorporating multiple ROs result in a substantial increase in area occupation [7]. To solve this problem, the proposed RO structure incorporates four customized inverter stages, enabling the system to operate at three distinct frequencies within a single RO structure. Having multiple operating frequencies allows TRNG to select the RO frequency that yields the highest level of randomness without increasing the number of RO structures.

In Figure 1a, the DFF “S1” functions as a frequency divider, accumulating jitter at the output “S1-Q” signal with a frequency equal to half of the RO output frequency. The second DFF “S2” generates the final output “S2-Q” by sampling the “S1-Q” signal. Figure 1b illustrates the timing diagram of the proposed RO-TRNG, comprising three stages: the RO, the jitter capturing, and the reset stages. In the RO stage, the TRNG initiates its operation by enabling the RO. The RO is activated by setting the “FF_Enable” signal to a logic high state. The jitter is accumulated during the activation of “FF_Enable”. The clock signal “S1-CLK” of the DFF “S1” is derived from the output of the RO. According to the RO output “S1-CLK”, when the rising edge is inserted, the output “S1-Q” is changed to its complementary logic state. Due to the jitter, the number of rising edges of the “S1-CLK” signal varies during each RO stage. When a sufficient amount of jitter is accumulated, the value of “S1-Q” becomes unpredictable and random. In the jitter-capturing stage, the “FF_CLK” signal becomes active to enable the DFF “S2”. The DFF “S2” samples the “S1-Q” final logic state and generates the final output “S2-Q” of the TRNG. Then, the TRNG moves to the reset stage by the negative trigger of the “FF_RST” signal, resetting the output values of both DFFs to the logic low state.

**Figure 2:**
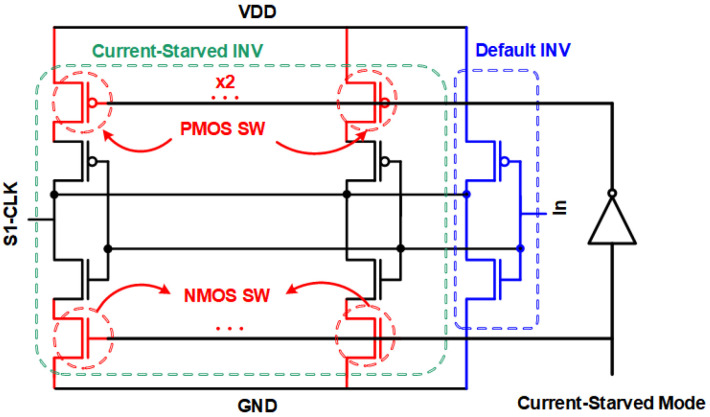
Schematic of the customized inverter.

Figure 2 shows the custom inverter structure, which incorporates four current-starved inverters in parallel with a default inverter. Frequency selection is performed by a “current-starved mode” (CSM) signal, which directly decides the operating conditions of the PMOS and NMOS switches (SW). These conditions include all switch transistors being turned on, turned off, and floating, leading to different output frequencies. The default inverter is required to guarantee proper operation of the RO, even in scenarios where the switches are off or floated.

When designing the proposed TRNG structure, the trade-off points always exist in achieving a balance between the randomness of the TRNG, power/energy consumption, and the area occupation [15]. This trade-off involves carefully optimizing the transistor sizing to meet the desired level of randomness while minimizing power/energy consumption and maintaining a reasonable area occupation. The design was simulated across various process corners—namely, FF (fast–fast), SS (slow–slow), and NN (normal–normal)—using a Monte Carlo simulation with 1000 sample points. In this context, FF, SS, and NN indicate the carrier mobility conditions of the NMOS and PMOS transistors, corresponding to fast, slow, and nominal mobilities, respectively. To assess the performance of the proposed structure more effectively, multiple designs with varying sizing configurations have been implemented in 28 nm processes.

## 3. Lightweight Photon Post-Processing for Proposed TRNG

NIST SP 800-22 is among the most well-known statistical tests used for evaluating the randomness of TRNGs. These standardized statistical tests comprise a total of 15 hypothesis tests, with certain tests encompassing various sub-tests [12]. It is challenging for any TRNG design to meet all these stringent statistical test requirements. A substantial number of state-of-the-art native TRNG designs have failed to satisfy the criteria of the NIST SP 800-22 statistical tests [9,16]. For the native TRNG designs that have been reported to successfully pass NIST SP 800-22 tests [6,17], it is noteworthy that the precise quantity of chips meeting the NIST SP 800-22 tests for these designs remains unspecified. This lack of information raises concerns about the overall performance and robustness of these designs, particularly when considering yield degradation problems. To address these concerns, NIST has released NIST SP800-90B [18], recommending the use of approved post-processing methods [19] to meet the NIST SP 800-22 requirements. NIST SP800-90B [18] provides comprehensive requirements for entropy sources essential for random bit generation (RBG), focusing on their role in ensuring randomness and security in cryptographic applications. NIST SP 800-90A recommends entropy source validation and proposes the amalgamation of entropy sources verified in 90B with verified post-processing techniques grounded in cryptographically secure primitives, such as block ciphers or hash functions, to enhance the randomness of RNG systems. However, these methods often come with high costs in area occupation and energy consumption, rendering them unsuitable for IoT devices. This section provides a concise analysis of two post-processing techniques considered to be lightweight in the literature [20]—namely, the XOR corrector and the Von Neumann corrector—as well as the vetted Deterministic Random Bit Generator (DRBG) mechanism, CTR-DRBG [19,21]. The analysis precedes an examination of the proposed Photon post-processing method, with the aim of identifying the most suitable lightweight post-processing algorithm that enhances TRNG randomness to meet the requirements of the NIST SP 800-22 test suite.

### 3.1. TRNG Post-Processing Candidates

#### 3.1.1. XOR Corrector

The XOR corrector performs the XOR operation on two consecutive TRNG blocks. The utilization of the XOR function takes the shape of an XOR tree to mitigate bias originating from the TRNG. The number of XOR stages employed to enhance the randomness of the TRNG output depends on the biased condition of the raw TRNG output. A larger bias in TRNGs will require more XOR stages to improve the randomness of the generated output. Due to its inherent simplicity, adding multiple XOR stages can be accomplished without substantially increasing area occupation. However, it is essential to acknowledge that this approach also results in a notable reduction, $D(1-1/2^{N})$, in the output rate of the TRNG system’s output rate, with D being the total number of the TRNG blocks and N being the number of XOR stages.

#### 3.1.2. Von Neumann Corrector

The Von Neumann corrector, which John von Neumann proposed in 1951 [22], can help to enhance the quality of random sequences generated by a TRNG. Von Neumann corrector takes two consecutive bits from a single TRNG and generates an output. Following the Von Neumann corrector operation, this algorithm can produce a perfect hamming weight [22]. However, this comes at the expense of a reduction in the output bit rate. Moreover, using the Von Neumann algorithm, an additional tracking block is incorporated to ensure a fixed output bit sequence [23].

#### 3.1.3. Ctr-Drbg with AES256

According to NIST [19], CTR-DRBG is a recommended post-processing algorithm that utilizes CTR-DRBG to generate multiple streams of random sequences by employing a single seed. However, the utilization of a single seed carries security risks, as it may eventually result in output repetition. Consequently, a reseed function becomes mandatory to validate the health of the seed after the generation of the output sequences. This inclusion, however, introduces complexity into the design and necessitates a larger area allocation. To utilize CTR-DRBG as a post-processing algorithm, CTR-DRBG generates only a single output sequence for each input seed. Consequently, in our evaluation, the reseed block has been omitted from consideration.

#### 3.1.4. Photon Hash Function Method

**Figure 3:**
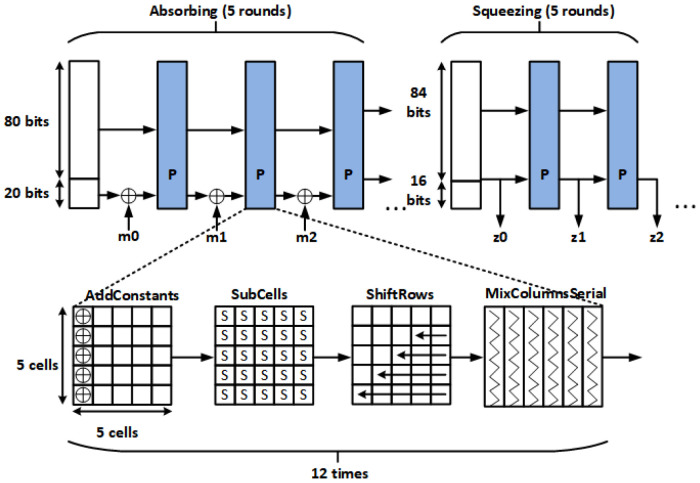
Photon80 abstract structure.

Photon is a cryptographic algorithm designed as a lightweight hash function and has been standardized by ISO/IEC [24]. Figure 3 illustrates the Photon structure, highlighting the Spongent framework and the integration of the AES-like primitive as the internal permutation state. The Photon permutation block adheres to the AES permutation state, comprising four sub-states: AddConstants, Subcells, ShiftRows, and MixcolumnsSerial. As the AES permutation state possesses a confusion characteristic capable of thoroughly scrambling input data to create an appearance of randomness, Photon serves as an efficient post-processing method to enhance the randomness of the outputs from the TRNG. Photon offers multiple output versions, including 80, 128, 160, 244, and 256. Among these versions, Photon80 is the smallest design. However, it is not recommended for hash applications due to its security vulnerability against collision attacks. Indeed, when Photon is employed solely as a post-processing algorithm for TRNGs, the resistance against collision attacks becomes redundant. In such cases, utilizing Photon80 as a post-processing algorithm can be a viable option to conserve area occupation.

### 3.2. Proposed Lightweight Photon Algorithms for TRNG Post-Processing

The discussed post-processing methods are evaluated in this section. The input dataset for evaluating the post-processing algorithm is collected from the implemented native TRNG. The NIST SP 800-22 results after post-processing are tested using the TRNG dataset on the XC7A200T field-programmable gate array (FPGA), and a comparison of the area and power consumption of these algorithms is conducted in the context of the 180 nm fabrication process. Table 1 summarizes the performance result of four post-processing algorithms.

In Table 1, the input bit file represents the minimum TRNG raw output needed for the post-processing algorithm to generate 100 M bits for testing with NIST SP 800-22 tests. From the summarized results in Table 1, the XOR corrector algorithm shows the best performance regarding area occupation and power consumption. However, the output sequence after applying the XOR corrector fails to pass the NIST SP 800-22 tests. It should be noted that Von Neumann requires a substantial number of input bits to generate a required output. In this experiment, the Von Neumann approach requires around 1200 M input bits to generate just 100 M output bits due to its operating principle. This huge demand for input bits ultimately increases energy consumption for the entire TRNG system. The Von Neumann corrector fails to mitigate the occurrences of the NIST SP 800-22 aperiodic bit pattern, consequently leading to a “non-overlapping test” failure. Unlike Von Neumann or XOR correctors, CTR-DRBG and Photon80 directly correct any correlation in the output of raw TRNGs. They subsequently scramble the output to minimize the occurrences of non-periodic bit patterns in the generated data. This improvement can be seen by the successful passing of the NIST SP 800-22 tests. Compared with CTR_DRBG, the implementation of Photon80 is five times smaller and consumes only one-third of the power, making it more suitable for resource-constrained devices. Based on our evaluation, Photon80 proves to be a suitable post-processing algorithm for the proposed TRNG structure, showcasing enhanced randomness levels, successful compliance with NIST SP 800-22 tests, and more efficient resource utilization compared to CTR-DRBG.

## 4. Implementation of the Proposed RO-TRNG with Lightweight Photon80 Post-Processing

**Figure 4:**
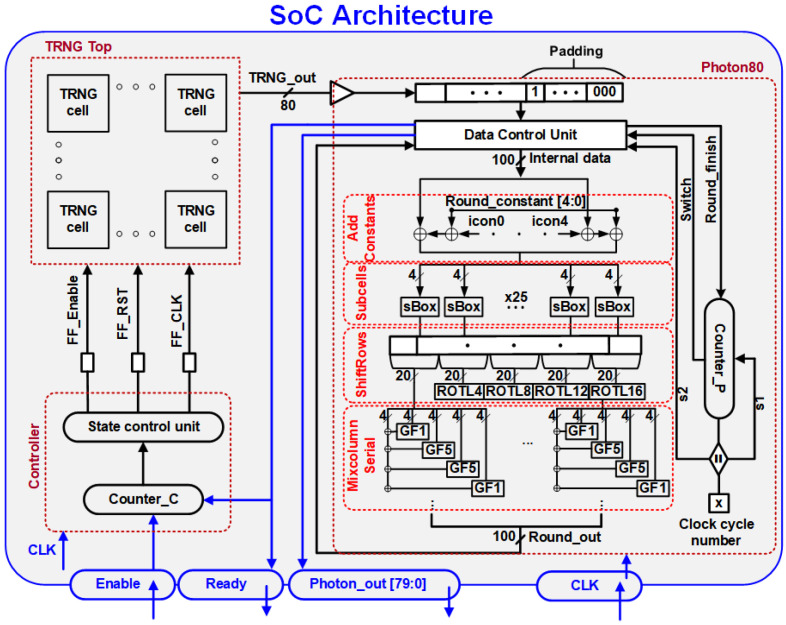
Proposed TRNGs with lightweight Photon80 post-processing architecture.

**Figure 5:**
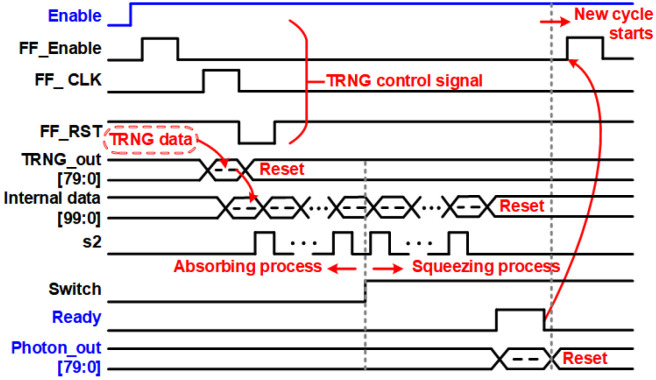
Timing diagram of the proposed TRNG with lightweight Photon post-processing.

Figure 4 and Figure 5 show the hardware architecture and timing diagram of the proposed TRNGs with the lightweight Photon80 post-processing. The system comprises three primary components: an 80-bit TRNG block, a Photon80 block, and a controller. The controller generates the necessary control signals for the TRNG block to generate its output by adhering to the timing diagram depicted in Figure 1. The controller becomes active under two conditions: either upon receiving the “Enable” signal when in the initial state or upon the activation of the “Ready” signal, initiating a new cycle to generate new “Photon_out” data. To monitor the operational phase and follow the timing diagram, a Counter_C block is utilized to track and control the activation of the “FF_Enable”, “FF_RST”, and “FF_CLK” signals. The TRNG core block is constructed by assembling 80 TRNGs, enabling the generation of an 80-bit sequence in a single operation. Subsequently, the TRNG output is utilized as the input for the Photon80 algorithm. The design of the Photon80 algorithm adheres to the operating principle outlined in [25]. The raw output of the 80-bit TRNG is padded with a single bit “1” followed by 19-bit “0”, resulting in a 100-bit padded input to the data control unit block. To process the 100-bit padded input, Photon80 performs five rounds of absorbing and five rounds of squeezing. In each absorbing round, 20 MSBs from the permutation output are XORed with a 20-bit message. During the squeezing state, 16 MSBs of the permutation output are extracted as the final output. This operational process is carried out by the data control unit block.

To generate the “Photon_out” signal, the permutation block needs to be executed 120 times. These execution times correspond to the five rounds of absorbing and five rounds of squeezing, with each round of absorbing/squeezing requiring 12 rounds of permutation. A counter, denoted as Counter_P block, is employed to keep track of the number of run-time operations of the permutation block by counting the clock cycles. It also communicates the current state, whether it is in the absorbing or squeezing state, to the data control unit. The “s1” signal aids the Counter_P block in counting the absorbing/squeezing rounds, while the “s2” signal informs the data control unit block when the permutation round is complete and updates the “internal data” signal. Once the absorbing stage is finished, a “switch” signal from the Counter_P block is exported to switch from the absorbing to the squeezing stage. The Photon round hardware structure and operation adheres to the design in our previous work [26].

After completing the Photon permutation round, the output signal “Round_out” is passed to the data control unit block, which generates the bit sequence for the subsequent round. Once the Photon80 completes its function and generates the output “Photon_out”, a “Ready” signal is generated. This “Ready” signal serves two purposes. Firstly, it notifies the Controller block to generate the necessary control signals for the next TRNG cycle, initiating the subsequent round of TRNG operation. Secondly, it serves as a notification to the measurement system that the output of the TRNG is about to be available for export.

## 5. Measurement Setup

**Figure 6:**
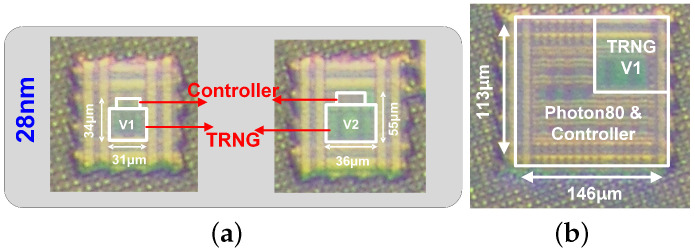
Chip micrograph of (**a**) the native TRNG in 28 nm processes and (**b**) 28_V1 TRNG design with lightweight Photon80 post-processing.

Figure 6a,b present the micrograph of the proposed TRNG, two native TRNG designs fabricated in a 28 nm process. Each TRNG design comprises a controller and 32 TRNG instances. The outputs of these 32 TRNG units are directly connected to the pad for measurement purposes. The size of the native TRNGs with the digital controllers are shown in Figure 6. Additionally, the SoC architecture includes Photon-based post-processing integrated with the native TRNG modules to improve randomness quality and enhance security, as illustrated in Figure 6b. Parallel measurement results for the version of 28 nm technology is presented to demonstrate the high performance of the implemented native TRNG structure. Finally, the measurement results of a best-performance native TRNG with Photon80 post-processing implemented in 28 nm technology are shown.

**Figure 7:**
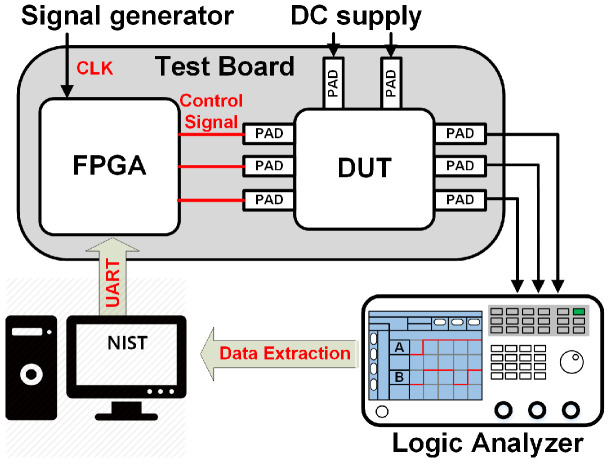
Measurement setup.

To evaluate the performance of the TRNG system, the TRNG outputs are extracted by following the setup in Figure 7. A signal generator generates the clock signal of the TRNG system. A DC supply provides a 1.0 voltage supply for 28 nm chips. The test bench is configured and loaded onto the FPGA. After programming, the control signals are generated from FPGA to activate the TRNG system. The TRNG output is obtained using a logic analyzer, which is then processed with MATLAB tested with [12,18] test suites. The chips are measured using the ring oscillator at three different frequencies: the maximum (CSM = VDD), minimum (CSM = VSS), and unidentified (floating CSM). These frequencies correspond to the conditions where all switch transistors are turned on (ON), turned off (OFF), and floating (FLO), respectively. It is important to note that, among the three measurement scenarios, if at least one scenario successfully passes the NIST tests [12,18], it is because the input CSM signal can be controlled easily. The clock frequency of 41.8 kHz, corresponding to a sampling interval of around 23 ns for the measurement, was chosen.

## 6. Measurement Results

### 6.1. Native TRNG Measurement Results

The proposed native TRNG is evaluated using 15 chip samples across 3 different test cases, totaling 18 tests. This approach ensures a thorough assessment of performance and robustness across various manufacturing processes.

**Figure 8:**
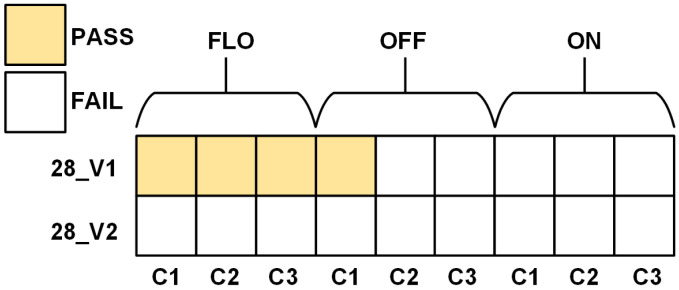
Measurement results of the native TRNGs with NIST SP 800-22 test suite.

Randomness is evaluated using the NIST SP 800-22 test suite, which is widely recognized as a comprehensive standard. The SP 800-22 test suite is performed on a one hundred million-bit sample measured from the implemented native TRNGs C1 to C3, with the chip number denoted from number one to number three. The measurement data were stored on a PC, and the test was conducted using the Linux operating system. Figure 8 shows the NIST SP 800-22 measurement results of implemented chips. Based on the evaluations of randomness, the 28_V1 design showed the best performance among the 28 nm CMOS designs. This is because both designs’ RO frequencies can be easily adjusted using the CSM signal. Table 2 summarizes the NIST SP 800-22 test results, demonstrating successful passing of all NIST SP 800-22 tests.

**Figure 9:**
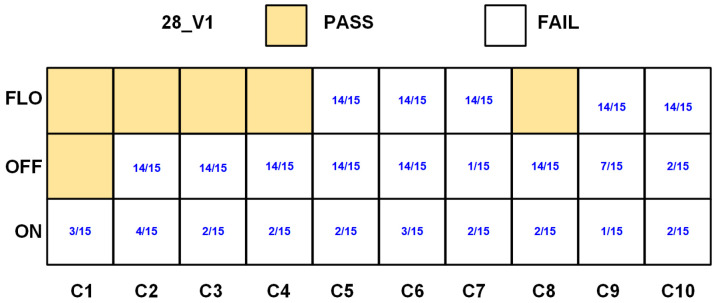
SP800-22 test results on 10 samples of the native TRNG (28_V1 chips).

The 28_V1 chip samples successfully pass all the NIST SP 800-22 tests while operating in the FLO configuration. To ensure the reliability of the measurements, the NIST SP 800-22 is conducted on a larger sample of 28_V1 chips. A notable impact of the variations in noise parameters is observed. This resulted in a decreased number of chips passing the NIST randomness criteria, as only 5 out of 10 samples successfully satisfied the SP800-22 requirements, as depicted in Figure 9, where C1 to C10 represent the chip sample numbers. The blue colored numbers denote the number of tests meeting the NIST SP 800-22 requirements over the 15 tests mentioned in [12]. Based on data measured from NIST SP800-22, the calculated Shannon entropy ranges from 0.999996 to 0.999999, indicating a high degree of randomness. The chips that did not pass the NIST evaluation failed the non-overlapping template matching test, which signals the presence of aperiodic bit patterns in the output sequences.

Table 3 compares the results of the proposed TRNG (28_V1) and previous works. The proposed TRNG structure implemented in the 28 nm process (28_V1) represents the smallest sized design, demonstrating competitive power consumption. It ranks as the second lowest power-consuming design among state-of-the-art TRNG structures. Among the 10 measured chips, only 5 passed the NIST SP800-22 tests. In contrast, other publications typically indicate only pass or fail without specifying the exact quantity, except for [11], which exclusively conducted tests on the NIST SP800-90B standard and did not mention tests from the SP800-22 suite.

### 6.2. Trng with Photon80 Post-Processing Measurement Results

Systematic yield issues are unavoidable in semiconductor manufacturing, particularly for circuits sensitive to noise parameters [29]. These variations can directly impact the number of chips that pass the NIST SP 800-22 test. To address variations in noise parameters and improve the yield rate, a post-processing algorithm is applied to the top-performing native TRNG design in the 28 nm process, specifically, the 28_V1. This integration aims to improve the noise parameters and enhance the overall quality of the TRNG output, thereby increasing the probability of passing all 15 NIST SP 800-22 tests for a greater number of chips.

Figure 6 depicts the micrographs of 80 TRNG blocks and the Photon80 lightweight post-processing algorithm implemented in a 28 nm CMOS process. The 28 nm chip has an area of 16,498 µm^2^, a throughput of 0.0142 Mbps, and a power consumption of 31.12 mW.

**Figure 10:**
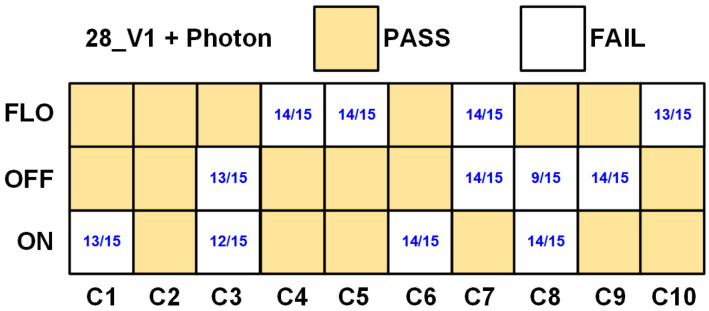
SP800-22 test results on 28_V1 chips with post-processing.

Figure 10 presents the results of NIST SP 800-22 tests of the native TRNG with the Photon80 post-processing algorithm. By applying the Photon80 post-processing algorithm, the number of chips passing the NIST SP 800-22 tests significantly increased by achieving a 100% yield rate (from 5/10 to 10/10), and the number of measured scenarios meeting the NIST randomness criteria increased by 55% (from 8/30 to 18/30). Table 4 summarizes the measurement results of the implementation, which includes 80 TRNG blocks and the Photon post-processing algorithm.

## 7. Conclusions

This paper proposed a new compact CMOS RO TRNG structure with a wave converter that converts the random jitter noise of an RO into a random output sequence. The proposed single RO TRNG can generate three different oscillation frequencies through the use of a multiple current-starved structure. This design allows for the selection of optimal performance among the three outputs by simply controlling a single gate input signal. The proposed native TRNGs are implemented in 28 nm processes. However, native TRNGs suffer from yield issues when testing large numbers of chip samples, as only 50% of the chip samples meet NIST SP 800-22 test requirements. A lightweight post-processing method utilizing the Photon80 hash function is proposed to address this issue. The proposed method effectively enhances the performance of the native TRNG structure, resulting in improved output randomness. Consequently, the number of TRNG samples that pass the NIST SP 800-22 tests increases by 50%. Additionally, this method has lower resource consumption in terms of area occupation and power consumption than the other vetted post-processing methods recommended in [18]. The prototype of the proposed TRNG, along with the implemented post-processing, successfully passes all NIST SP 800-22 tests across all measured chip samples. The design occupies 16,498 µm^2^ in 28 nm technology, with a throughput of 0.0142 and power consumption of 31.12 at the nominal 1.0 V.

## Figures and Tables

**Table 1:** Performance comparison of TRNG post-processing candidates.

	XOR Corrector	Von Neumann Corrector	CTR_DRBG (AES256)	Photon80
Area [GE]	431	4403	64,829 (x)	13,915 (0.22x)
Power [mW]	1.8	20.6	99.2 (y)	34.4 (0.35y)
Input bit file [Mbit]	200	1200	150	100
NIST SP 800-22 test suite	Fail	Fail	Pass	Pass

**Table 2:** Native TRNG with NIST SP 800-22 statistical tests.

Statistical Tests	*p*-Value	Pass?
Frequency (Monobit) Test	0.27	Yes
Frequency Test within a Block	0.49	Yes
Runs Test	0.46	Yes
Test for the Longest Run of Ones in a Block	0.23	Yes
Binary Matrix Rank Test	0.79	Yes
Discrete Fourier Transform (Spectral) Test	0.33	Yes
Non-overlapping Template Matching Test	0.49	Yes
Overlapping Template Matching Test	0.91	Yes
Maurer’s (Universal Statistical) Test	0.59	Yes
Linear Complexity Test	0.30	Yes
Serial Test	0.41	Yes
Approximate Entropy Test	0.76	Yes
Cumulative Sums (Cusum) Test	0.36	Yes
Random Excursions Test	0.611	Yes
Random Excursions Variant Test	0.55	Yes

**Table 3:** Summary of the native TRNG measurement results and comparison with state-of-the-art design.

	ISSC2014 [6]	ISSCC2017 [27]	JSSC2017 [17]	ISSCC2021 [28]	ISSCC2024 [11]	This Work
Technology (nm)	28	65	65	14	4	28
Entropy source	Jitter	Jitter	Metastability and Jitter	Metastability	Jitter	Jitter
Structure	3-edge RO	Differential RO	Sense-amp	Latch	Self-Timed Ring	Waive Con. RO
Area (µm^2^)	375	920	1609	2114	1289	74 ^a^
Measured Voltage (V)	N/A	1.08	1.2	0.65	0.675∼0.935	1
Power (mW)	0.54	0.289	5	3.7	N/A	0.433
# Samples pass NIST SP 800-22	N/A ^b^	N/A ^b^	N/A ^b^	N/A ^b^	N/A ^b^	5/10
Post-processing	No	No	No	Yes	No	No

^a^ TRNG core occupied 30 µm^2^, controller occupied 44 µm^2^, total area is 74 µm^2^. ^b^ indicates the TRNG passed all 15 NIST tests.

**Table 4:** TRNG with Photon 80 Post-Processing Performance Summary.

Technology	Area (µm^2^)	Power (mW)	# Samples Pass NIST SP 800-22
28 nm	16,498	31.12	All pass (10/10)

## Data Availability

The original contributions presented in the study are included in the article, further inquiries can be directed to the corresponding author.
